# Gross national happiness and health: lessons from Bhutan

**DOI:** 10.2471/BLT.15.160754

**Published:** 2015-08-01

**Authors:** Gyambo Sithey, Anne-Marie Thow, Mu Li

**Affiliations:** a Sydney School of Public Health, Edward Ford Building, The University of Sydney, New South Wales, 2006, Australia.; b Menzies Centre for Health Policy, University of Sydney, Sydney, Australia.

Bhutan was the first country in the world to pursue happiness as a state policy. The Bhutanese concept of happiness is deeper than the common meaning of happiness in industrialized countries. The philosophy of gross national happiness has several dimensions: it is holistic, recognizing people’s spiritual, material, physical or social needs; it emphasizes balanced progress; it views happiness as a collective phenomenon; it is both ecologically sustainable, pursuing well-being for both current and future generations, and equitable, achieving a fair and reasonable distribution of well-being among people.^1^ Since the early 1970s, Bhutan has promoted population well-being over material development. Happiness, health and well-being are closely related.^2^^,^^3^ Good health is often considered the single most important determinant of well-being;^1^^,^^2^ conversely, adverse health changes have lasting and negative effects on well-being.^4^

In industrialized countries, happiness is often linked with material consumption. A basic level of material wealth is necessary, but citizens of richer and more technologically advanced countries are not necessarily the happiest.^5^ Along with economic growth, there is a need to measure well-being and ecological sustainability to reflect the overall progress of nations and of humankind.^6^ Given increasing evidence that the current trajectory of human development is not sustainable, there is an urgent need for more inclusive measures of progress than traditional economic indicators such as gross domestic product.^7^

Since the global recession of 2008–2009, the importance of well-being has gained political momentum – driven, in part, by a perception that the poorest and most vulnerable members of society are paying the price for excessive greed and risk-taking in the financial sector. In Europe, a shift in emphasis from measuring economic production to measuring people’s well-being has been recommended.^7^^,^^8^ Following a resolution proposed by Bhutan, the United Nations convened a high level meeting at which the Secretary-General Ban Ki-moon called for development outcomes that value and measure happiness and well-being.^9^

The recent political momentum and the close links between health and well-being present an opportunity for health objectives to be included in other policy domains. Gross national happiness has greatly influenced the health system in Bhutan, as reflected in the constitution which states that “the state shall provide free access to basic public health services in both modern and traditional medicines.” Health is recognized as a prerequisite for economic and spiritual development and as a means to achieving gross national happiness.^10^

In Bhutan, 7.4–11.4% of total government spending is in the health sector.^11^ Primary health care is emphasized; privatization of health services is prohibited. A health trust fund was established in 1998 to ensure uninterrupted supply of essential drugs and vaccines. These policies are based on the philosophy of gross national happiness and provide an indication of the population-health benefits of prioritizing well-being in national policy-making. Bhutan screens all sectoral plans and policies to ensure that they are consistent with gross national happiness.

At the global level, translating gross national happiness into policy has the potential to promote health as defined in World Health Organization (WHO) charter, acknowledging the role of the environment, ecological sustainability, good governance and social determinants. WHO can play a more active role in strengthening consultation between sectors, improving access to relevant data and disseminating evidence on health and well-being. As a leader in the happiness movement, Bhutan has hosted several international conferences on gross national happiness; a further conference will be held in November 2015.

The conference will be an opportunity to collate and disseminate the latest evidence from Bhutan and other countries linking health and well-being. Participants will discuss tools needed to pursue research and policy initiatives that contribute to sustainable development goals. First, the philosophy of gross national happiness needs to be understood more widely in the corporate boardroom. Second, the required indicators should be incorporated into current databases in the health sector. Third, the health sector has a responsibility to communicate the fact that health, human happiness and ecologically sustainable development are interdependent. 
